# Optimization Design of Mix Proportion for Fly Ash–Silica Fume–Basalt Fiber–Polypropylene Fiber Concrete under Steam Curing Condition

**DOI:** 10.3390/ma17091971

**Published:** 2024-04-24

**Authors:** Ziqian Li, Gang Li, Chong Wang, Wei Li, Huaping Zheng

**Affiliations:** 1 College of Water and Architectural Engineering, Shihezi University, Shihezi 832000, China; 17699539020@163.com; 2 State Key Laboratory of Frozen Soil Engineering, Northwest Institute of Eco-Environment and Resources, Chinese Academy of Sciences, Lanzhou 730000, China; wangchong@lzb.ac.cn; 3 Xinjiang Golden Land Cement Products Co., Ltd., Shihezi 832000, China; 17799430013@163.com (W.L.); 17799431377@163.com (H.Z.)

**Keywords:** orthogonal experiment, steam-cured concrete, pore characteristic, microstructure

## Abstract

To enhance the physical and mechanical characteristics of steam-cured concrete, an orthogonal experimental design was utilized to examine the effects of varying contents of fly ash (0 wt%, 10 wt%, 15 wt%, 20 wt%), silica fume (0 wt%, 5 wt%, 10 wt%, 15 wt%), basalt fiber (0 vol%, 0.05 vol%, 0.1 vol%, 0.2 vol%), and polypropylene fiber (0 vol%, 0.05 vol%, 0.1 vol%, 0.2 vol%) on its mechanical properties. Utilizing range and variance analyses, this study identified four preliminary optimized compositions of concrete incorporating fly ash, silica fume, basalt fiber, and polypropylene fiber. On this basis, in order to determine the optimal mix proportion, the mechanical performances, the pore characteristics, and the microstructure of four optimized mix proportions were analyzed. According to the results of macroscopic, fine, and microscopic multi-scale tests, the addition of 15 wt% fly ash, 10 wt% silica ash, 0.2 vol% basalt fiber, and 0.1 vol% polypropylene fiber to the steamed concrete is the best to improve the performance of the steamed concrete. Compared to ordinary concrete, the compressive strength increases by 28%, the tensile strength increases by 40%, and the porosity decreases by 47.2%.

## 1. Introduction

Cast-in-place concrete has certain drawbacks including low efficiency, high consumption, and significant labor intensity [1]. Moreover, it contributes to issues such as dust pollution, noise pollution, and urban environmental pollution. In contrast, precast concrete technology offers several advantages over the cast-in-place method. It not only enhances the construction efficiency and the quality of concrete components but also effectively reduces waste generation and noise pollution [2]. As a result, precast concrete components are extensively utilized in various fields including bridges, tunnels, highways, and railways [3,4]. Therefore, precast concrete components have become an important means of achieving industrialization within the construction industry [5,6].

In the manufacture of precast concrete elements, steam curing emerges as the predominant curing technique utilized. This approach enhances equipment utilization rates, abbreviates the production timeline, and diminishes construction expenses [7]. Although steam curing can accelerate the hydration rate of cement and improve the early strength of concrete, it will also have adverse effects on precast components. Too high steaming temperature and too long steaming time affect the strength improvement of the later stage of steaming concrete and reduce the impermeability of concrete, while too low steaming temperature and too short steaming time will reduce the release strength of concrete [8], resulting in poor durability and service performance of concrete structures [9,10]. To mitigate the adverse effects associated with steam curing, researchers have enhanced the constrained durability of concrete through the incorporation of mineral admixtures. The pozzolanic reactions and microaggregate filling attributes of mineral admixtures refine the pore architecture and diminish the permeability of concrete [11]. Zeyad et al. [12] and Moser et al. [13] have shown that mineral admixtures possess the ability to reduce the quantity of large pores as well as the total porosity in concrete, consequently diminishing the diffusion capacity of chloride ions within the concrete matrix. Ali et al. [14] showed that silica fume (SF) has been found to enhance the compressive strength of concrete at the 7th, 28th, and 90th day of curing, and it not only improves the transition zone of the concrete interface but also makes the microstructure of concrete pores more dense. The introduction of slag and fly ash (FA) into the mix can attenuate the initial strength of concrete, and it bolsters the durability of steam-cured concrete through microstructural enhancement [15,16]. In addition to silica fume and fly ash, nanomaterials can also be used to refine the pore structure of concrete. For example, carbon nanotubes (CNTs) can reduce the total porosity, refine the pore structure, improve the mechanical properties of concrete, delay crack propagation, and accelerate cement hydration by acting as a nucleation seeding site for C-S-H, and CNTs also have high thermal stability [17]. The performance of concrete can also be improved by the addition of fibers, and basalt fibers (BFs) and polypropylene fibers (PFs) have been extensively incorporated and analyzed in steam-cured concrete due to their superior properties and cost-effectiveness. Zhang et al. observed that the incorporation of basalt fibers (BFs) notably improves the flexural strength of steam-cured concrete, achieving an increase of as much as 14% relative to traditional concrete [18]. Additionally, it markedly enhances the crack resistance toughness of concrete. Yang found that the simultaneous inclusion of steel fibers (SFs) and polypropylene fibers (PFs) in steam-cured concrete resulted in compressive and bending strengths superior to those of both ordinary concrete and concrete with a single type of fiber [19]. Wang and He et al. demonstrated that hybrid fiber (BF-PF)-reinforced concrete exhibits higher compressive strength and a greater corrosion resistance coefficient than concrete containing only basalt fibers (BFs) or only polypropylene fibers (PFs) at 7, 14, and 28 days [20,21].

Despite extensive research into the individual effects of fly ash (FA), silica fume (SF), basalt fibers (BFs), and polypropylene fibers (PFs) on steam-cured concrete, the collective impact of these components on the material’s performance remains underexplored. Therefore, the effects of FA, SF, BF, and PF content on mechanical properties, pore characteristics, and microstructure of concrete are studied based on orthogonal tests. Based on the results of multi-scale tests, the optimum content of fly ash, silica ash, basalt fiber, and polypropylene fiber was determined.

## 2. Experimental Materials and Methods

### 2.1. Materials

**Figure 1:**
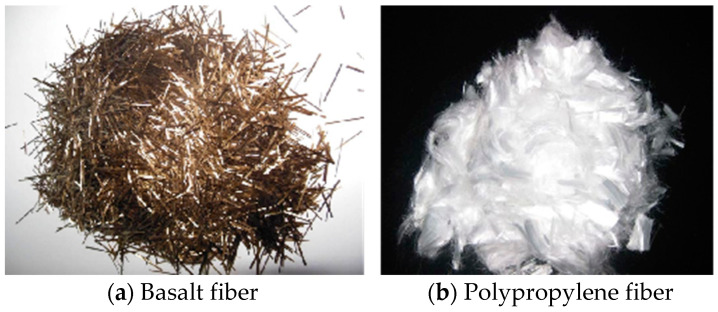
Appearance of fibers.

The cement used is PO 42.5 ordinary Portland cement, and its parameters are shown in Table 1. The parameters of FA and SF used are listed in Table 2 and Table 3, respectively. The chosen fine aggregate is medium sand, distinguished by a fineness modulus of 2.7. For coarse aggregate, pebbles are used, featuring a maximum particle size of 31 mm. Table 4 lists the parameters of the BF and PF used, and their appearances are shown in Figure 1.

### 2.2. Design of Orthogonal Experiment

The orthogonal experiment is an experimental design method that involves multiple factors and levels of research. Its principle lies in selecting representative and typical points from numerous experiments for analysis, aiming to achieve efficiency, speed, and economy [22]. In this study, C50 concrete is selected as the research object, and the orthogonal test is employed to design combinations of different contents for FA, SF, BF, and PF. To identify the optimized mix proportions, range and variance analyses were performed on the experimental data concerning compressive and splitting tensile strength. The orthogonal test adopted a four-factor, four-level design, as shown in Table 5, with the orthogonal experiment design outlined in Table 6. A, B, C, and D represent four different factors, namely FA, SF, BF, and PF, respectively. The numerals 1, 2, 3, and 4 represent four distinct dosages of these factors. For instance, A1B2C3D4 refers to contents of FA, SF, BF, and PF of 0 wt%, 5 wt%, 0.1 vol%, and 0.2 vol%, respectively.

### 2.3. Sample Preparation and Mechanical Test

**Figure 2:**
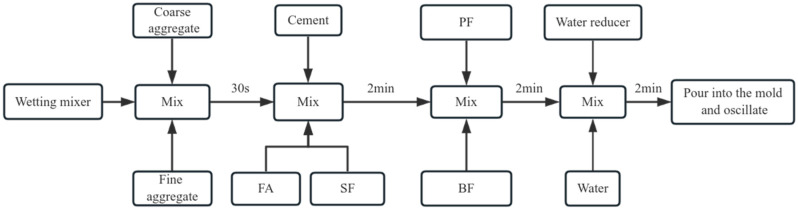
Sample preparation process.

**Figure 3:**
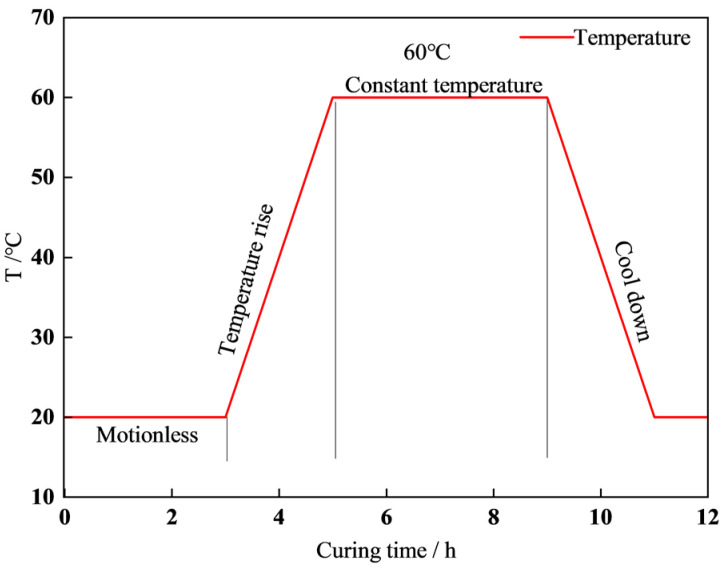
Steam curing setting.

The process for preparing the concrete is depicted in Figure 2. After molding, the samples were positioned in a steam curing chamber for steam curing. The concrete curing system is shown in Figure 3. The compressive and splitting tensile strength tests were conducted in compliance with the relevant standards (GB 50081-2019 [23], GB 50107-2010 [24], and JGJ/T 221-2010 [25]). For each set of tests measuring concrete compressive strength and split tensile strength, three cube specimens measuring 100 mm × 100 mm × 100 mm are prepared. The cube compressive strength loading rate should be 0.5–0.8 MPa/s, while the split tensile strength loading speed should be 0.05–0.08 MPa/s. The strength value of the final specimen is calculated as the arithmetic average of the measured values of the three specimens. If the difference between the maximum or minimum value of the three measured values and the median value exceeds 15% of the median value, the maximum and minimum values are excluded, and the median value is taken as the strength value of the specimen. As the specimen is non-standard, with dimensions of 100 mm × 100 mm × 100 mm, the cube compressive strength and split tensile strength results must be converted to standard specimen results using size conversion factors. The cube compressive strength size factor is 0.95, and the split tensile strength size factor is 0.85. And the test time nodes were as follows: (1) end of steam curing (0d); (2) standard curing for 7 days after steam curing (7d); (3) standard curing for 28 days after steam curing (28d).

### 2.4. Nuclear Magnetic Resonance (NMR) Test

**Figure 4:**
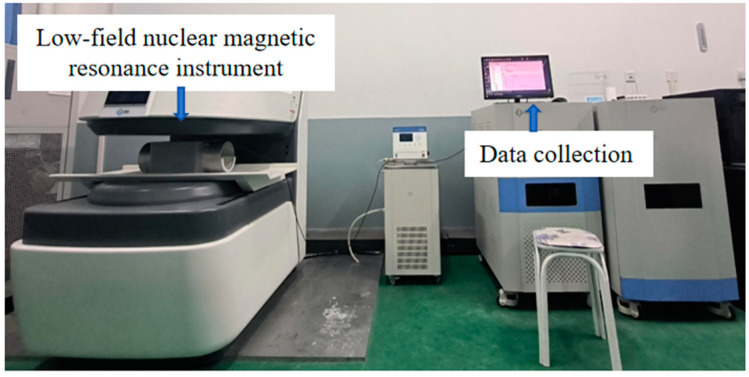
Low-field NMR analysis system.

In this research, the pore attributes of the concrete specimens were assessed via the Macro MR12-150H-I low-field nuclear magnetic resonance (NMR) analysis system manufactured by Macro, Suzhou, China. The equipment is shown in Figure 4.

Nuclear magnetic resonance is principally employed for investigating the internal structural characteristics of porous materials, with its foundational principle being the depiction of the sample’s pore information via the relaxation time (*T*_2_). The correlation between the relaxation time and pore size can be articulated as follows [26]:

(1) $$r=F_{s}\rho_{2}T_{2}$$

where r is the pore size (μm), $F_{s}$ is the geometric shape factor of the pore structure, and $\rho_{2}$ is the transverse surface relaxation strength (μm·ms^−1^).

### 2.5. Scanning Electron Microscope (SEM) Test

The SEM test was carried out using a Su-8010 SEM scanner manufactured by Hitachi, Tokyo, Japan. The concrete specimens were made into specimens with a diameter of about 5 mm, soaked in anhydrous ethanol for 24 h, and then taken out, dried and sprayed with gold, and placed under the SEM to observe their microstructures. In order to further quantitatively analyze the microstructure changes in the samples, Image-Pro Plus (IPP) was used to identify the pores in the microstructure and show them in green.

## 3. Orthogonal Experimental Results Analysis

The experimental outcomes regarding the mechanical properties of concrete are documented in Table 7. To assess the effect of FA, SF, BF, and PF on the compressive and splitting tensile strength, range and variance analyses were conducted on the compressive strength of concrete at 0, 7, and 28d and on the splitting tensile strength at 7 and 28d. The outcomes of the analysis are delineated in Table 8, Table 9, Table 10 and Table 11. Range analysis, also known as intuitive analysis, is based on the assumption that if we focus on factor A, we can assume that the influence of other factors on the result remains the same, so that the change in the different levels of factor A is mainly caused by itself. The range reflects the change in the influence of the factor levels on the test results. ANOVA, also known as “analysis of variance” or “F-test”, essentially examines the effect of categorical independent variables on numerical dependent variables [27].

### 3.1. Compressive Strength

Table 8 displays the range analysis findings for the compressive strength of concrete samples at 0, 7, and 28d. The ranking of the four factors’ influence on compressive strength at 0d is determined to be SF > FA > BF > PF, while for compressive strength at 7 and 28d, the sequence is SF > BF > FA > PF. These results were obtained by comparing the range (R) values of the various factors.

**Figure 5:**
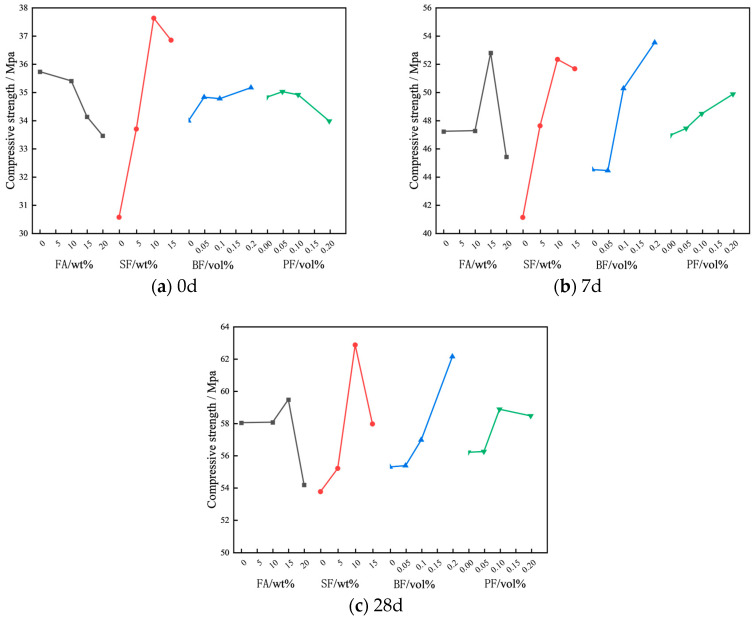
Effect of different factors on the compressive strength of concrete.

Figure 5 depicts the impact of various factors on the compressive strength of concrete. Figure 5 reveals that the compressive strength of concrete follows diverse patterns as the content of FA increases. When the curing age is 0d, a discernible decrease in the compressive strength of concrete is observed with an increment in FA content. Specifically, with the FA content rising from 0 wt% to 20 wt%, there is the decrease in compressive strength by 6.7%. This phenomenon can be attributed to the decreased reactivity of FA, resulting in a reduced degree of hydration during the initial stages of cementitious material formation [28]. At the curing ages of 7d and 28d, the compressive strength of concrete demonstrates a pattern of initial enhancement followed by a decrease as the FA content escalates. It is noteworthy that the optimal compressive strength of concrete is achieved at a fly ash concentration of 15 wt%. As the SF content increases, the compressive strength of concrete at 0, 7, and 28d exhibits the pattern of initially increasing and subsequently decreasing. Specifically, when the SF content reaches 10 wt%, the compressive strength at 0d, 7d, and 28d reaches its peak values of 37.63, 52.33, and 62.87 MPa, respectively. The factors contributing to the enhancement of concrete compressive strength through the analysis of SF and FA are as follows: (1) because of their small particle size, SF and FA possess a microaggregate effect and can effectively fill the structure of concrete; (2) both FA and SF demonstrate a pozzolanic effect and can react with the calcium hydroxide Ca(OH)_2_ generated during cement hydration to produce calcium silicate hydrate (C-S-H) gel. This gel contributes to further compacting the concrete structure. Therefore, due to the combined effects of microaggregation and the volcanic ash effect, the compressive strength of concrete is enhanced.

As for volume percentage of BF increases from 0 vol% to 0.2 vol%, there is a corresponding enhancement in the compressive strength of concrete. When the BF content is 0.2 vol%, compared to ordinary concrete without BF, the compressive strength improves by 3.4% at 0d and by 20.3% and 12.24% at 7 and 28 days, respectively. The enhancement in compressive strength can be attributed to the capability of BF to inhibit pore connectivity, disrupt the formation of larger pores, and refine the overall pore structure, thereby contributing to the material’s improved mechanical properties [29]. However, for PF, its influence on the compressive strength of concrete is not significantly apparent. With the increase in PF content, the change in compressive strength is observed to be less than 6% [30].

Table 9 presents the outcomes of the variance analysis conducted on the compressive strength data. Based on the results of the variance analysis, the impact of SF, FA, BF, and PF on enhancing the compressive strength of concrete decreases progressively at 0d, with SF emerging as the most significant factor. The compressive strength of concrete at 7 days is significantly affected by SF, FA, and BF. Specifically, SF exerts the most substantial influence, followed by BF and FA, whereas the impact of PF content on the 7-day compressive strength is not significant. Furthermore, SF, FA, BF, and PF exhibit some impact on the 28-day compressive strength of concrete, although it is not significant. The above results are in agreement with those of range analysis. Therefore, combining the results of range and variance analyses, the optimized mix proportion of concrete can be preliminarily obtained as A1B3C4D2, A3B3C4D4, and A3B3C4D3.

### 3.2. Splitting Tensile Strength

Table 10 showcases the results of the range analysis performed on the splitting tensile strength. The results reveal that, at 7d, the splitting tensile strength is most influenced by BF, followed by SF, PF, and FA, whereas at 28d, the influence order is BF > PF > SF > FA. These results were obtained by comparing the range (R) values of the various factors.

**Figure 6:**
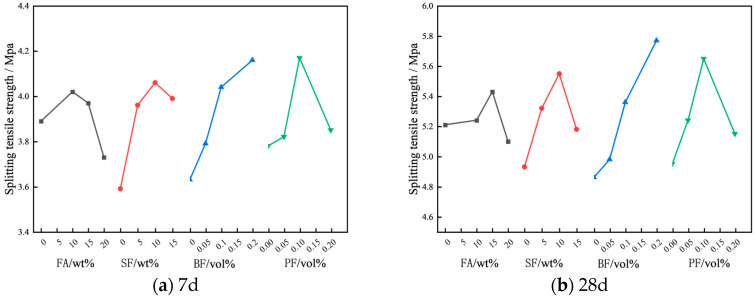
Effect of different factors on the splitting tensile strength of concrete.

Figure 6 illustrates the impact of various factors on the splitting tensile strength of concrete. From Figure 6, it is evident that the splitting tensile strength of steam-cured concrete initially increases, followed by a decrease as the FA content increases. At 7d the splitting tensile strength of the concrete, when mixed with 10 wt% FA, attains its maximum value, registering a 7.7% increase over that of ordinary concrete. At the age of 28d, incorporating 15 wt% fly ash into the concrete mixture results in a maximal enhancement of splitting tensile strength, achieving an increase of 6.4%. Derived from the aforementioned results, it is evident that FA does not significantly influence the splitting tensile strength of concrete. The presence of a specific quantity of FA exerts a relatively modest enhancement on the splitting tensile strength of concrete. However, surpassing this threshold may diminish the enhancing effect and potentially lead to a negative impact on the splitting tensile strength. This phenomenon can largely be ascribed to the considerable replacement of cement by FA, which leads to a decrease in the hydration products of cement, consequently diminishing the splitting tensile strength of concrete. As the SF content escalates, the splitting tensile strengths of concrete at 7 days and 28 days exhibit a trend of initial augmentation followed by a subsequent diminution. Furthermore, the optimal content of SF at both 7d and 28d is 10 wt%, resulting in respective increases of 13% and 16.9% in splitting tensile strength. This phenomenon occurs due to the microaggregate impact and the pozzolanic reaction of SF, which serve to densify the concrete’s structure, thereby enhancing its splitting tensile strength.

The splitting tensile strength of concrete at 7d and 28d rises in correlation with an increase in BF content (0.05~0.2 vol%). After adding 0.2 vol% BF, the splitting tensile strength at 7d and 28d increases by 14.6% and 18.7%, respectively. The splitting tensile strength of concrete shows an initial enhancement and then a reduction with the increasing PF content, pinpointing the optimal PF concentration at 0.1 vol%. The findings indicate that an appropriate quantity of PF content enhances the splitting tensile strength of concrete. However, an excessive incorporation of PF induces a detrimental mixing effect, ultimately resulting in a diminished splitting tensile strength of the concrete. This is primarily because the optimal concentration of PF can be uniformly distributed within the concrete and bond with the cementitious materials, yielding a specific crack resistance effect that consequently enhances the concrete’s splitting tensile strength. However, if the PF content is too high, agglomeration will occur in the concrete, which not only reduces the utilization rate of PF but also reduces the bond strength between the fibers and the concrete [31], resulting in a reduction in the splitting tensile strength of the concrete [32].

Table 11 depicts the variance analysis undertaken for the splitting tensile strength of concrete. The findings reveal that BF constitutes the most critical factor affecting the splitting tensile strength at 28 days, whereas other factors exert some impact on the 28-day splitting tensile strength of concrete, albeit insignificantly. Based on the range analysis of the splitting tensile strength, the optimal mix proportions for steam-cured concrete have been identified as A2B3C4D3 and A3B3C4D3.

The results of the above orthogonal experimental analysis show that the optimized mix proportions based on compressive strength are: A1B3C4D2, A3B3C4D4, and A3B3C4D3; while the optimized mix proportions based on splitting tensile strength are: A2B3C4D3 and A3B3C4D3.

## 4. Determination of Optimal Mix Proportion

To identify the optimal mix proportion from the four initially optimized mix proportions, this research evaluated and analyzed the macroscopic mechanical properties, pore characteristics, and microstructure of the 28-day samples of both the four optimized mix proportion concretes and the normal concrete (NC).

### 4.1. Macroscopic Mechanical Performances

**Figure 7:**
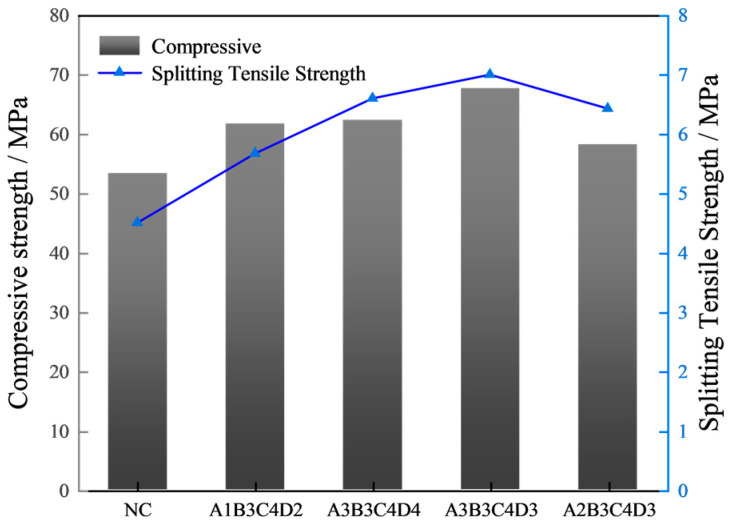
Compressive strength and splitting tensile strength.

Figure 7 displays the experimental outcomes for compressive and splitting tensile strength of concrete across five mix proportions, measured at 28 days. It is obvious that compared with ordinary concrete, the four optimized mix proportions significantly improve the compressive and splitting tensile strength of concrete. Among them, the mix proportion of A3B3C4D3 has the greatest effect on improving the mechanical performances of concrete. Relative to ordinary concrete, there is an increase in compressive strength by 28% and in splitting tensile strength by 40%. Therefore, based on the experimental results of macroscopic mechanical performances, the optimal mix proportion of steam-cured concrete can be determined as A3B3C4D3 (FA = 15 wt%, SF = 10 wt%, BF = 0.2 vol%, and PF = 0.1 vol%).

### 4.2. Pore Characteristics

**Figure 8:**
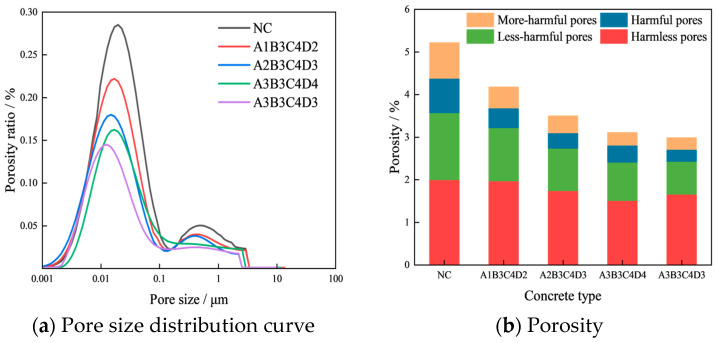
Pore characteristics.

Figure 8a presents the pore size distribution curves for five different types of concrete. The most probable pore sizes for samples NC, A1B3C4D2, A2B3C4D3, A3B3C4D4, and A3B3C4D3 are 0.022798, 0.017247, 0.015, 0.01702, and 0.013045 μm, respectively. The above results show that the pore structure of concrete is optimized by four kinds of optimal mix ratio, with the A3B3C4D3 mix proportion exhibiting the most effective refinement of the concrete’s pore size. Figure 8b displays the porosity results of the samples. The porosities of NC, A1B3C4D2, A2B3C4D3, A3B3C4D4, and A3B3C4D3 samples are 5.1%, 4.08%, 3.5%, 3.01%, and 2.69%, respectively. Consequently, the internal densification achieved with the four optimized concrete mix proportions surpasses that of ordinary concrete. With the mix proportion set to A3B3C4D3, the concrete exhibits the highest density in its internal structure. According to the classification of concrete pore structure [33]: harmless pores (HMLPs) < 0.02 μm, less harmful pores (LHMFPs) 0.02~0.05 μm, harmful pores (HMFPs) 0.05~0.2 μm, and more harmful pores (MHMFPs) > 0.2 μm. Figure 8b also lists the pore composition of the concrete sample. The contents of LHMFPs, HMFPs, and MHMFPs in the samples of A1B3C4D2, A2B3C4D3, A3B3C4D4, and A3B3C4D3 are lower than those in the sample of NC, indicating that the pore composition of concrete is enhanced by all four optimized mix proportions. In addition, the contents of LHMFPs, HMFPs, and MHMFPs in the sample of A3B3C4D3 are the least. Therefore, from the analysis of pore composition, it can also be deduced that the mix proportion of A3B3C4D3 has the optimal effect in optimizing the pore structure of the concrete.

**Figure 9:**
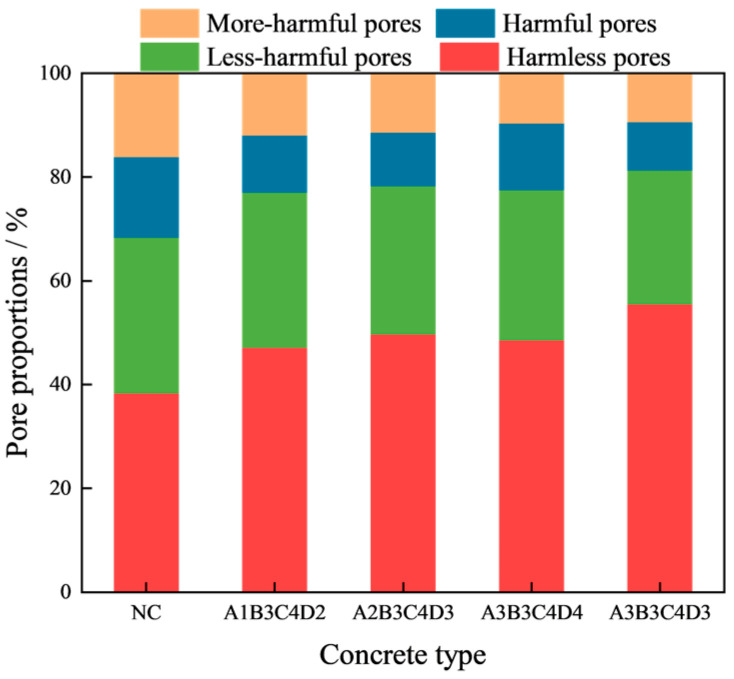
Pore proportion of concrete samples.

To further analyze the influence of four optimized mix proportions on the pore composition, we calculated the proportions of HMLPs, LHMFPs, HMFPs, and MHMFPs in the porosity, as shown in Figure 9. The proportions of LHMFPs, HMFPs, and MHMFPs in the porosity of samples A1B3C4D2, A2B3C4D3, A3B3C4D4, and A3B3C4D3 are observed to be lower compared to those of NC. This suggests an enhancement in the pore composition of the concrete due to the utilization of the four optimized mix proportions. Furthermore, for the concrete mix with proportions A3B3C4D3, the proportions of LHMFPs, HMFPs, and MHMFPs are the most minimized. Therefore, based on the results of pore proportion, it can be inferred that A3B3C4D3 has the best refining impact on the pore structure of concrete.

Upon analyzing the most probable pore size, porosity, pore content, and pore distribution, we have reached the following conclusions: all four optimized mix proportions (A1B3C4D2, A2B3C4D3, A3B3C4D4, and A3B3C4D3) can optimize the pore structure of concrete, and A3B3C4D3 (FA = 15 wt%, SF = 10 wt%, BF = 0.2 vol%, and PF = 0.1 vol%) has the best optimization effect on the pore characteristics of concrete.

### 4.3. Microstructure

**Figure 10:**
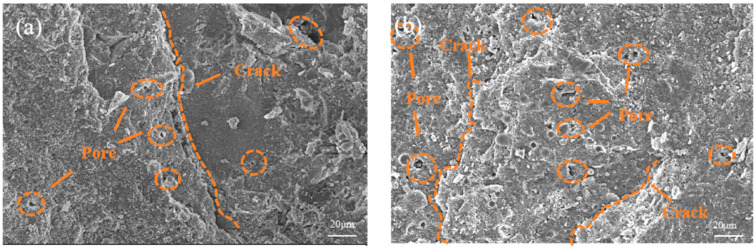

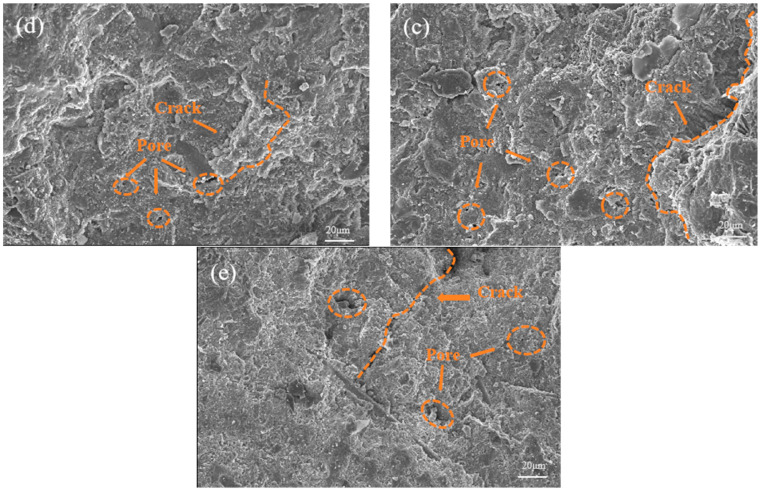
Microstructures of concrete samples: (**a**) NC; (**b**) A1B3C4D2; (**c**) A2B3C4D3; (**d**) A3B3C4D3; (**e**) A3B3C4D4.

To select the optimal mix proportion from the perspective of microstructure among the four preliminary optimized mix proportions, we conducted an SEM test, and the findings are presented in Figure 10.

The microstructural analysis of five concrete samples reveals that the normal concrete (NC) specimen, depicted in Figure 10a, exhibits the highest porosity, largest pore sizes, and widest cracks. A qualitative examination of the microstructures suggests that the four optimized mix proportions (A1B3C4D2, A2B3C4D3, A3B3C4D4, and A3B3C4D3) significantly improve the concrete’s microstructure. This enhancement is attributed to the inclusion of FA, SF, BF, and PF in these mixes. The synergistic interaction between the microaggregate effect of FA and SF and the volcanic ash effect, coupled with the crack-resistance bridge effect of BF and PF, results in a denser concrete microstructure [34,35].

**Figure 11:**
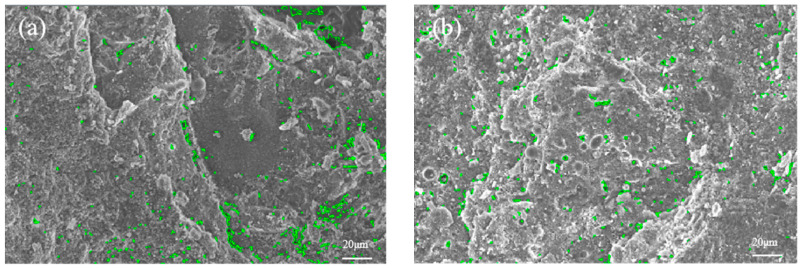

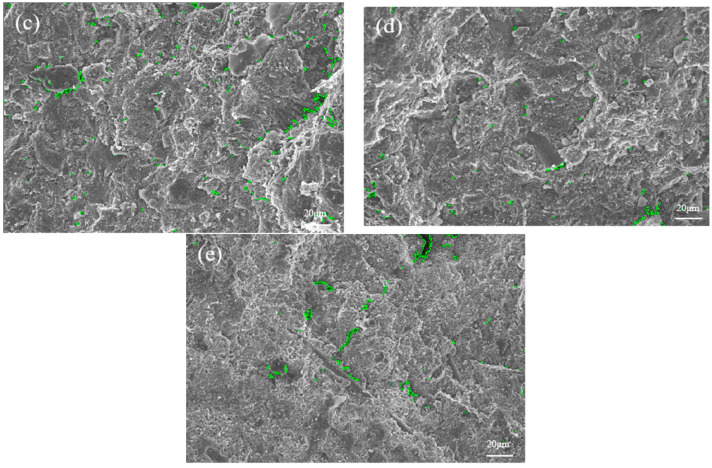
Pores of microstructure (×500): (**a**) NC; (**b**) A1B3C4D2; (**c**) A2B3C4D3; (**d**) A3B3C4D3; (**e**) A3B3C4D4.

Figure 11 shows the pore proportion of the microstructure, the green colour in the figure shows the porous part of the microstructure of Figure 10. Furthermore, the minimum pore area (Min-PA), the maximum pore area (Max-PA), and the porosity were calculated as well (Equation (2) [36]), with the results presented in Table 12.

(2) $$P=\frac{A_{p}}{A}\times 100\%$$

where P is porosity (%), *A* stands for the total area of the microstructure (μm^2^), and *A_p_* denotes the total area of the pores (μm^2^).

Microstructure scanning imagery, acquired at a 500× magnification, exhibits statistical deviations in pore characteristics relative to the comprehensive sample analysis conducted via nuclear magnetic resonance. Furthermore, the IPP software (Version 6.0.0.260) imposes a constraint, resulting in a uniform Min-PA across all images [36]. Consequently, the analysis is confined to the Max-PA and porosity metrics. According to Table 12, the porosities of NC, A1B3C4D2, A2B3C4D3, A3B3C4D3, and A3B3C4D4 are 2.85%, 2.25%, 2.06%, 0.71%, and 1.09%, respectively. Meanwhile, the maximum pore areas of NC, A1B3C4D2, A2B3C4D3, A3B3C4D3, and A3B3C4D4 are 32.70, 13.96, 10.82, 7.84, and 22.41 μm^2^, respectively. The porosity and maximum pore area of the four optimized mix proportions are lower compared to those of conventional concrete. Thus, the optimization of four mix proportions is capable of refining the pore structure and augmenting the densification of concrete. In addition, the sample with the mix proportion of A3B3C4D3 exhibits the smallest Max-PA and porosity among the five types of concrete samples. Hence, from the quantitative microstructural analysis, it is concluded that with the concrete mix proportion A3B3C4D3 (FA = 15 wt%, SF = 10 wt%, BF = 0.2 vol%, and PF = 0.1 vol%), the concrete possesses the densest microstructure.

## 5. Conclusions

(1) Range and variance analyses reveal the sequence of impact of four components (FA, SF, BF, and PF) on the 28d compressive strength of steam-cured concrete as SF > BF > FA > PF. Moreover, the sequence of their effect on the 28d splitting tensile strength of steam-cured concrete is determined as BF > PF > SF > FA.
(2) Optimal performance in FA-SF-BF-PF steam-cured concrete is attained with the constituent ratios of 15 wt% for FA, 10 wt% for SF, 0.2 vol% for BF, and 0.1 vol% for PF.
(3) Upon incorporating the optimal proportions of FA, SF, BF, and PF into steam-cured concrete, the 28d compressive and splitting tensile strengths of the concrete demonstrate respective enhancements of 28% and 40% in comparison to conventional concrete.
(4) Autoclaved concrete has the best pore characteristics and densest microstructure using the optimal blend of fly ash, silica fume, basalt fiber, and polypropylene fiber, resulting in a 47.2% reduction in porosity compared to normal concrete.

## Figures and Tables

**Table 1:** Parameters of cement.

Density/ g·cm^−3^	Time/min		Flexural Strength/MPa		Compressive Strength/MPa	
	Initial Setting	Final Setting	3d	28d	3d	28d
3.1	178	228	6.4	9.2	30.6	57.3

**Table 2:** Parameters of fly ash.

Grade	Fineness/%	Loss on Ignition/%	Water Demand Ratio/%	Moisture Content/%
I	7.7	2.1	94	0.3

**Table 3:** Parameters of silica fume.

SiO_2_/%	Loss on Ignition/%	Specific Surface Area/m^2^·g^−1^	Cl^−^/%
96.2	3.92	19.1	0.07

**Table 4:** Fiber parameters.

Types	Length/mm	Diameter/μm	Density/g·cm^−3^	Elongation/%	Tensile Strength/MPa	Elasticity Modulus/GPa
BF	12–15	7–15	2.65	2.8	4000	105
PF	12	35.86	0.91	25	3700	35.86

**Table 5:** Factors and levels of mix proportion design.

Level	FA/wt%	SF/wt%	BF/vol%	PF/vol%
	A	B	C	D
1	0	0	0	0
2	10	5	0.05	0.05
3	15	10	0.1	0.1
4	20	15	0.2	0.2

**Table 6:** Design of orthogonal experiment (kg·m^−3^).

Number	Water	Cement	Coarse Aggregate	Sand	FA	SF	Water Reducer	BF	PF
1	172	490	1128	610	0	0	8.33	0	0
2	172	449	1128	610	41	0	8.33	1.32	0.455
3	172	428.5	1128	610	61.5	0	8.33	2.65	0.91
4	172	408	1128	610	82	0	8.33	5.3	1.82
5	172	469.5	1128	610	0	20.5	8.33	1.32	0.91
6	172	416	1128	610	41	20.5	8.33	0	1.82
7	172	408	1128	610	61.5	20.5	8.33	5.3	0
8	172	388	1128	610	82	20.5	8.33	2.65	0.455
9	172	449	1128	610	0	41	8.33	2.65	1.82
10	172	408	1128	610	41	41	8.33	5.3	0.91
11	172	387	1128	610	61.5	41	8.33	0	0.455
12	172	367	1128	610	82	41	8.33	1.32	0
13	172	428.5	1128	610	0	61.5	8.33	5.3	0.455
14	172	388	1128	610	41	61.5	8.33	2.65	0
15	172	367	1128	610	61.5	61.5	8.33	1.32	1.82
16	172	346.5	1128	610	82	61.5	8.33	0	0.91

**Table 7:** Results of orthogonal test.

Number	FA	SF	BF	PF	Compressive Strength/MPa			Splitting Tensile Strength/MPa		
					0d	7d	28d	0d	7d	28d
1	1	1	1	1	32.00	35.85	52.38	2.78	3.10	4.5
2	2	1	2	2	31.20	36.83	53.12	2.70	3.17	4.598
3	3	1	3	3	30.50	48.80	56.12	2.50	3.77	5.885
4	4	1	4	4	28.50	44.90	53.34	2.10	3.00	5.72
5	1	2	2	3	34.50	42.70	52.26	3.10	3.60	4.94
6	2	2	1	4	33.17	45.40	54.08	3.27	3.90	5.1
7	3	2	4	1	33.20	56.50	62.71	3.65	4.12	6.57
8	4	2	3	2	33.87	45.83	51.70	3.10	4.05	5.57
9	1	3	3	4	42.03	55.30	66.03	3.90	4.10	5.472
10	2	3	4	3	37.50	56.70	68.08	3.67	4.67	6.77
11	3	3	1	2	36.13	52.00	58.67	2.96	3.45	4.848
12	4	3	2	1	36.87	45.30	55.69	2.80	3.80	5.1
13	1	4	4	2	38.90	56.03	61.48	3.50	4.30	6.93
14	2	4	3	1	37.20	51.10	54.00	3.15	3.80	4.53
15	3	4	2	4	36.70	53.85	60.38	2.70	4.10	5.3
16	4	4	1	3	34.60	45.70	56.00	2.93	3.60	4.67

**Table 8:** Range analysis results of compressive strength.

	Level	0d				7d				28d			
		A	B	C	D	A	B	C	D	A	B	C	D
K	1	35.73	30.55	33.98	34.82	47.22	41.09	44.49	46.94	58.04	53.74	55.28	56.2
	2	35.4	33.69	34.82	35.02	47.26	47.61	44.42	47.42	58.07	55.19	55.36	56.24
	3	34.13	37.63	34.77	34.91	52.79	52.33	50.26	48.47	59.47	62.87	56.96	58.87
	4	33.46	36.85	35.16	33.97	45.43	51.67	53.53	49.86	54.18	57.96	62.15	58.46
Optimum level		1	3	4	2	3	3	4	4	3	3	4	3
R		2.27	7.08	1.18	1.06	7.36	11.23	9.11	2.93	5.29	9.13	6.87	2.67

**Table 9:** Variance analysis results of compressive strength.

Compressive Strength	Factor	Degree of Freedom	Sum of Squares	Mean Square	F	p
0d	A	3	13.594	4.531	2.074	0.282
	B	3	125.892	41.964	19.21	0.018 *
	C	3	3.006	1.002	0.459	0.731
	D	3	2.789	0.93	0.426	0.749
	Error	3	6.553	2.184	−	−
7d	A	3	122.201	40.734	33.556	0.008 **
	B	3	319.544	106.515	87.747	0.002 **
	C	3	242.949	80.983	66.714	0.003 **
	D	3	20.141	6.714	5.531	0.097
	Error	3	3.642	1.214	−	−
28d	A	3	70.017	23.339	0.949	0.485
	B	3	107.607	35.869	1.459	0.178
	C	3	155.407	51.802	2.106	0.276
	D	3	29.396	9.799	0.398	0.759
	Error	3	73.779	24.593	−	−

Note: * significant; ** highly significant.

**Table 10:** Range analysis results of splitting tensile strength.

Factor	Level	7d				28d			
		A	B	C	D	A	B	C	D
K	1	3.89	3.59	3.63	3.78	5.21	4.93	4.86	4.95
	2	4.02	3.96	3.79	3.82	5.24	5.32	4.98	5.24
	3	3.97	4.06	4.04	4.17	5.43	5.55	5.36	5.65
	4	3.73	3.99	4.16	3.85	5.1	5.18	5.77	5.15
Optimum level		2	3	4	3	3	3	4	3
R		0.29	0.47	0.53	0.39	0.33	0.62	0.91	0.7

**Table 11:** Variance analysis results of splitting tensile strength.

Splitting Tensile Strength	Factor	Degree of Freedom	Sum of Squares	Mean Square	F	p
7d	A	3	0.268	0.062	0.278	0.84
	B	3	0.16	0.183	0.818	0.564
	C	3	8.452	0.228	1.02	0.494
	D	3	0.379	0.127	0.568	0.673
	Error	3	1.005	0.223	−	−
28d	A	3	0.222	0.089	0.267	0.847
	B	3	0.805	0.053	0.16	0.917
	C	3	2.014	2.817	8.407	0.047 *
	D	3	1.05	0.126	0.377	0.778
	Error	3	1.579	0.335	−	−

Note: * significant.

**Table 12:** Pore characteristics of microstructure.

Number	Minimum Pore Area/μm^2^	Maximum Pore Area/μm^2^	Porosity/%
NC	0.47259	32.70	2.85
A1B3C4D2	0.47259	13.96	2.25
A2B3C4D3	0.47259	10.82	1.09
A3B3C4D3	0.47259	7.84	0.71
A3B3C4D4	0.47259	22.41	2.06

## Data Availability

Data are contained within the article.
